# Acute Aortic Occlusion Presenting as Flaccid Paraplegia

**DOI:** 10.1155/2015/713489

**Published:** 2015-03-11

**Authors:** Ayman Kilany, Jasem Y. Al-Hashel, Azza Rady

**Affiliations:** ^1^Department of Neurology, Ibn Sina Hospital, P.O. Box 25427, Safat, 13115 Kuwait City, Kuwait; ^2^Department of Research on Children with Special Needs, National Research Center, Egypt

## Abstract

A 67-year-old male known to be hypertensive and diabetic had a sudden onset of severe low back pain and flaccid paraplegia with no sensory level or bladder affection and the distal pulsations were felt. Acute compressive myelopathy was excluded by MRI of the dorsal and lumbar spines. The nerve conduction study and CSF analysis was suggestive of acute demyelinating polyneuropathy. The patient developed ischemic changes of the lower limb and CT angiography revealed severe stenosis of the abdominal aorta and both common iliac arteries. We emphasize the importance of including acute aortic occlusion in the differential diagnosis of acute flaccid paraplegia especially in the presence of severe back pain even if the distal pulsations were felt.

## 1. Introduction

Acute aortic occlusion (AAO) is an uncommon vascular emergency that can present with predominantly neurologic symptoms due to spinal cord ischemia. It frequently causes mortality unless urgent and appropriate diagnosis is followed immediately by proper management [1]. Two primary causes were identified: embolism (65%) and thrombosis (35%). Heart disease and female gender were risk factors for embolism, while hypertension, smoking, and diabetes were risk factors for thrombosis [2]. The clinical presentation may vary from acute limb ischemia, neurological symptoms of the lower extremities, abdominal symptoms, and acute hypertension [3]. Clinicians must have a high index of suspicion in patients who present painful paresis or paraplegia. Clinical examination of peripheral pulses in these patients is mandatory [4]. Initial failure to diagnose aortic occlusion, with an intermediate delay from presentation to diagnosis of 24 hours, is mainly responsible for bad prognosis. Even after diagnosis had been established, the need for urgent revascularization was not always recognized, the time from diagnosis to revascularization being 13 hours. However, the diagnosis may evade detection since collateral vasculature can maintain a basal perfusion and prevent the expression of acute ischemic phenomena for a long time [5].

We are reporting a case that demonstrates the difficulty of reaching early accurate diagnosis because of the numerous etiologies that may present with similar symptoms.

## 2. Case Presentation

Our case is a sixty-seven-year-old male known to be heavy smoker, hypertensive, poorly controlled diabetic on oral hypoglycemic agents and have long standing ischemic heart disease and history of lymphoma. The patient presented complaining of intense lower back pain, numbness, and severe weakness in both legs. Physical examination revealed severe and almost symmetrical paraplegia with areflexia. However, he had no clear sensory level deficit and no point tenderness on the thoracic or lumbar spine. The lower extremities had intact, but weak, dorsalis pedis pulses bilaterally. The patient also had no signs of upper extremity motor or sensory deficit. He was otherwise awake, alert, and oriented to person, place, time, and situation.

An urgent magnetic resonance imaging without contrast of the thoracic and lumbar spine showed a widely patent spinal canal with no cord compression or intrinsic abnormality of the cauda equina.

**Figure 1:**
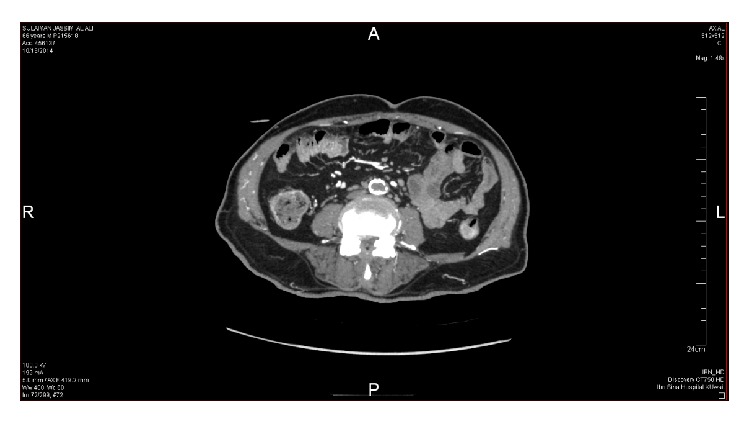
Thrombosis and calcification of the abdominal aorta on computed tomography with angiographic study (axial view).

**Figure 2 fig2:**
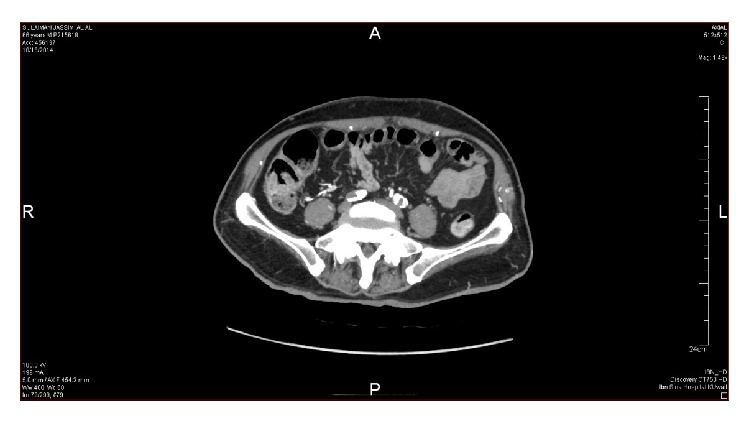
Occlusion and calcification of common iliac arteries were observed (axial view).

**Figure 3:**
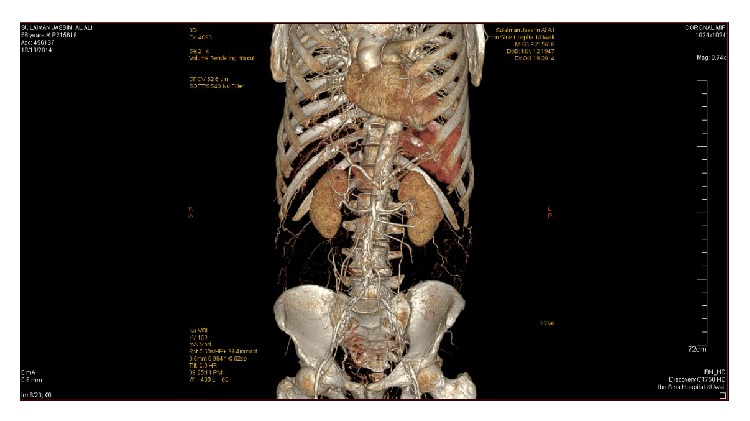
Thoracoabdominal computed tomography with angiographic study showing thrombosis of the distal aorta and both common iliac arteries (coronal view).

Nerve conduction study done twice revealed prolonged distal latencies and decreased conduction velocity of both lower limbs with prolonged F-wave latency and bilaterally absent H-reflex suggestive of demyelination and axonal damage. A lumbar puncture was performed and the CSF study showed elevated protein 764 mg/dL with normal cells and glucose. The working diagnosis initially was acute demyelinating polyneuropathy and a course of IVIG was started. The patient did not improve and continued to complain of severe low back pain; therefore, we decided to look for possible malignancy especially with the available history of lymphoma. Tumor markers, CT chest, pelvis, and lumbosacral spines were done. CT chest was suggestive of lymphangitis carcinomatosis and B2-microglobulin was significantly elevated (5.2 mg/L). A computed tomographic arteriogram of the abdomen, pelvis, and lower extremities was about to be performed, but the patient developed typical chest pain with ECG changes and elevated cardiac troponin. An urgent coronary angiogram was performed through a radial puncture and two coronary stents were placed. Within 2 days after discharge from CCU, we noticed discoloration of the right big toe. Immediately a computed tomographic arteriogram of the abdomen, pelvis, and lower extremities revealed severe stenosis of the abdominal aorta, 25 mm above the bifurcation, and both common iliac arteries (Figures 1 [Fig fig2]–3). The patient immediately referred to surgical ICU for further management. The offered management was in the form of bilateral above knee amputation, which the patient refused, or a trial for endovascular embolectomy with very high risk of death due to reperfusion syndrome. Within few hours the patient developed severe acidosis and septicemia with acute renal failure. The general condition of the patient continued to deteriorate despite aggressive antimicrobial and renal replacement therapy and died 72 hours later.

## 3. Discussion

The clinical syndrome of acute paraplegia is caused by traumatic spinal cord compression, ischemic spinal cord injury resulting from occlusion of the aorta or supplying arteries, or by spinal cord compression caused by a hematoma or empyema. Aortic occlusion has been known to occur among patients with heart and/or atherosclerotic aortoiliac disease [6]. AAO is a rare—most of the available literatures are case reports of nonsurgical patients—but catastrophic event with 75% mortality and 20–50% even after revascularization [7]. This syndrome can be mistaken for neurologic disorders and is missed in up to 50% of cases presenting with paraplegia [8]. In our patient, incomplete occlusion has left the distal pulses intact, which further obscured and delayed the diagnosis. In AAO, paraplegia is caused by occlusion of the aorta either above or below the level of the artery of Adamkiewicz, leading to serious cord ischemia and infarction or causing ischemia of the peripheral nerves and musculature distal to the occlusion, respectively [9]. Although the patient had no formal history for thrombophilia, he did have a history of cancer in the past that was treated with chemotherapy which may predispose him to thrombosis in addition to ischemic heart disease and calcified aortoiliac axis.

Spontaneous aortic occlusion typically presents with intense ischemic pain and a profound systemic response, including tachycardia, diaphoresis, and a mottling of the extremities. The classic five P's, pain, pallor, pulselessness, paralysis, and paresthesias, can be diagnostic clues and CT angiography remains the gold standard modality of diagnosis [10].

In their clinical study of 18 cases of aortic occlusion, Littooy and Baker [11] reported a mean interval of about 18 hours from the onset of symptoms to definitive treatment. They reported a high perioperative mortality rate (40–62.5%). Various complications, including renal failure, compartment syndrome, adult respiratory distress syndrome, myocardial infarction, and disseminated intravascular coagulation, were also reported [5]. In more recent series of 49 reported patients with acute aortic thrombosis, 14 presented with primary neurological deficit. Of those patients, nine died and five survived. The procedures performed in survivors included three aortic tube graft reconstructions, one aortobifemoral bypass, and one endovascular stent placement. Once the diagnosis is made, anticoagulation should be immediately initiated and urgent revascularization procedures including thromboembolectomy, aortic reconstruction, anatomic or extra-anatomic bypass, and thrombolysis. The choice of approach depends on etiology, anatomy, and patient factors [7].

From this case, we stress the importance of considering AAO in the differential diagnosis of acute paraplegia especially in the presence of severe pain at onset. The auscultation of the iliac arteries and measurement of ankle-brachial pressure index are reliable and simple tools for early detection of AAO. Also, we emphasize the paramount importance of CT angiography to exclude AAO, even in the presence of distal pulsations, in those cases.
